# Analysis of Multi-Layer RNA Modification Patterns for the Characterization of Tumor Immune Microenvironment in Hepatocellular Carcinoma

**DOI:** 10.3389/fcell.2021.761391

**Published:** 2021-11-10

**Authors:** Jiyuan Xing, Shen Shen, Zihui Dong, Xiaobo Hu, Lixia Xu, Xiaorui Liu, Qinggang Li, Yize Zhang, Gangying Cui, Zujiang Yu

**Affiliations:** ^1^ Gene Hospital of Henan Province, Precision Medicine Center, The First Affiliated Hospital of Zhengzhou University, Zhengzhou, China; ^2^ Department of Infectious Diseases, The First Affiliated Hospital of Zhengzhou University, Zhengzhou, China

**Keywords:** hepatocellular carcinoma, RNA methylation modification, TME, immunotherapy, prognosis

## Abstract

**Background:** RNA modifications have emerged as important posttranscriptional changes in multiple tumor cellular processes and tumorigenesis, including hepatocellular carcinoma (HCC). However, the potential roles and the interaction between regulators of RNA modifications and the tumor microenvironment (TME) are unclear in HCC.

**Methods:** The gene expression profiles of 26 RNA modification “writers” were investigated in the TCGA cohort. The unsupervised clustering approach was used to class these RNA modification regulators. The characteristics of immune cell infiltration from TME for each cluster was tested by the CIBERSORT method. Additionally, we established a scoring model to evaluate the RNA modification characteristics of individual tumors. The associations between the scoring model and genetic as well as clinical characteristics, drug sensitivity, and response to immunotherapy were also analyzed.

**Results:** We mapped the somatic mutations and somatic copy number variation of the RNA modification regulators. The expression of all selected regulators was detected, and two modification patterns were identified that featured distinct immune cell infiltration characteristics. Subsequently, we developed a score model (termed as WM-Score model). Furthermore, the survival analysis showed that the WM-Score value was associated with HCC patient prognosis. The results of the ROC curves analysis and multivariate analysis all confirmed that the WM-Score value was strongly associated with anti-cancer drug resistance and therapeutic efficacy of immunotherapy, thus could be used as an independent risk factor in HCC.

**Conclusion:** Our research identified two RNA modification patterns characterized by distinct TME, and the WM-Score model was developed that might serve as reliable prognostic and immunotherapeutic effect predictor of HCC.

## Introduction

In 2018, hepatocellular carcinoma (HCC) was predicted to be the sixth most prevalent cancer worldwide (Bray et al., 2018), with a 5 years survival rate as low as 9.1%, and an overall median survival of 9 months (Giannini et al., 2015). Infection by HBV or HCV, chronic alcohol consumption, and obesity-related NASH are the principal causes of HCC (Llovet et al., 2021). The condition is usually diagnosed at an advanced stage, therefore, effective treatments for advanced metastatic HCC are limited. Although there are surgical and chemotherapy options, the mortality rate of HCC remains high. Forms of immunotherapy, such as immune checkpoint inhibitors (ICIs) have been used to capture the disease progression and to enhance adaptive immunity in advanced HCC (Ou et al., 2020). Meanwhile, only a subset of patients show therapeutic response to ICIs, and this response it is difficult to predict. Therefore, a deeper understanding of the molecular mechanism of HCC is necessary to improve patient survival.

Recently, RNA modifications, coined the “epitranscriptome”, have emerged as crucial posttranscriptional regulators of the gene expression process (Barbieri and Kouzarides, 2020). Increasing evidence has revealed that these modifications have huge implications for human pathophysiology, including cancer (Frye et al., 2016; Jonkhout et al., 2017; Nachtergaele and He, 2017; Ontiveros et al., 2019). Accordingly, over 170 different types of chemical modifications of cellular RNAs have been described, among which methylation modifications account for two-thirds and are widely present in various RNA types (Barbieri and Kouzarides, 2020). The most abundant and better characterized internal RNA modification is N^6^-methyladenosine (m^6^A) that regulates multiple aspects of RNA metabolism, such as RNA processing, RNA translation, and nuclear export (Roundtree et al., 2017; Sun et al., 2019). N1-methyladenosine (m1A) is an important post-transcriptional RNA modification that has been found in tRNA, rRNA, mitochondrial RNA and mRNA (RajBhandary et al., 1966; Peifer et al., 2013; Li et al., 2017; Safra et al., 2017). APA is an RNA-processing mechanism that generates distinct 3′ termini on mRNAs and other RNA polymerase II transcripts (Tian and Manley, 2017). RNA editing mediated by adenosine deaminase acting on RNA enzymes a well-documented post-transcriptional mechanism altering nucleotide in selected transcripts (Nishikura, 2010). RNA modification is catalyzed by RNA methyltransferases called “writers” (they add a specific modification), demethylases or “erasers” (they remove a specific modification), and m^6^A-binding proteins or “readers” (they recognize and bind modified nucleotides). The RNA modification is a dynamic process, and the interaction between each type of methylation modification has not yet been fully elucidated (Davalos et al., 2018; Xue et al., 2020; Nombela et al., 2021).

Accumulating evidence supports the prominent role of the complex and diverse tumor immune microenvironment (TIME), including cancer cells, locally infiltrating immune cells, stromal cells, and active medium, in tumor cell proliferation, invasion, and metastasis (Azambuja et al., 2019; Fu et al., 2019). Non-malignant cells are not only one of the major players of cancer progression, but also determine the immunotherapeutic response (Lu et al., 2019). Therefore, a comprehensive analysis of the diversity of TME and different immune phenotypes can guide and improve immunotherapeutic responsiveness (Binnewies et al., 2018).

In this study, we focused on the most heavily modified RNA types, including m^6^A, alternative polyadenylation (APA), m^1^A, and A-to-I RNA editing. Furthermore, we comprehensively analyzed the correlation between various types of RNA modification regulators and cell-infiltrating characteristics of TIME by integrating the genomic and transcriptomic alterations of samples from The Cancer Genome Atlas - Liver Hepatocellular Carcinoma (TCGA-LIHC) databases. Two distinct modification patterns with different immune cell characteristics were identified. In addition, we developed the WM-Score model to quantify the efficacy of “writers” in modifying individual tumors and to predict the prognosis and immunotherapeutic response of HCC patients.

## Methods

### Data Acquisition and Processing

The gene expression profiles and clinical annotations were downloaded from the Cancer Genome Atlas (TCGA) portal (http://cancergenome.nih.gov/). Data cohorts with missing information were removed. A total of 356 cases of TCGA-LIHC were used for further analysis. The R Bioconductor package and R (version 3.6.2) were employed for data analysis.

Drug sensitivity data were collected from The Genomics of Drug Sensitivity in Cancer (GDSC) database (www.cancerRxgene.org) (Yang et al., 2013). Spearman’s correlation analysis was utilized to evaluate the association between the scoring model and drug reaction, where |Rs| > 0.2, and FDR <0.05 was considered significant correlation.

The immunotherapy dataset IMvigor210 cohort was used to explore the immunotherapy response and prognosis of HCC patients with different WM-Score values. The standardized RNA-sequencing data of 1111 HCC patients with detailed clinicopathological data were downloaded from http://research-pub.gene.com. The data were analyzed using the IMvigor210CoreBiologies R package.

### Unsupervised Clustering Analysis

In order to explore the robust clustering of HCC cases, we employed the unsupervised clustering approach to analyze the gene profiles of RNA modification writers. A total of 26 RNA modification regulators, including seven m^6^A modification enzymes (KIAA1429, METTL14, ZC3H13, METTL3, WTAP, RBM15B, and RBM15), 12 APA modification enzymes (CPSF1, CPSF2, CPSF3, CPSF4, CSTF1, CSTF2, CSTF3, CF1, PCF11, CLP1, NUDT21, and PABPN1), four m^1^A modification enzymes (TRMT10C, TRMT6, TRMT61A, and TRMT61B), and three A-I modification enzymes (ADARB1, ADARB2 and ADAR) were analyzed. An NMF-based consistent clustering algorithm was used to determine RNA modification patterns based on the mRNA expression of analyzed regulators. Unsupervised cluster analysis was performed by The Consensus Cluster Plus package as previously described (Wilkerson and Hayes, 2010).

### Gene Set Variation Analysis (GSVA)

GSVA is a gene set enrichment method that provides increased power to estimate changes of subtle pathway activity over a sample population in an unsupervised manner (Hänzelmann et al., 2013). We conducted GSVA analysis to explore the association between RNA modifications and biological processes. The gene set “h.all.v7.2” and “c2.cp.kegg.v7.1”were derived from the MSigDB database (Zhu et al., 2020). The functional annotation of 26 “writer” genes was conducted by the clusterProfiler R package, with a cutoff value of FDR <0.05. An adjusted P with value < 0.05 was considered as indicative of statistical significance.

### Cell-type Identification by Estimating Relative Subsets of RNA Transcripts (CIBERSORT)

CIBERSORT is a method that can accurately estimate the fraction of diverse cell subsets in gene expression profiles from complex tissues (http://cibersort.stanford.edu) (Newman et al., 2015). To predict the immune subset composition of HCC samples from gene expression profiles, CIBERSORT was used to estimate the relative abundance of 22 types of immune cells (model = absolute, permutation = 1,000, disable quantile normalization for RNA-Seq data as recommended).

### Construction of the WM-Score Scoring System

Firstly, the RNA modification-related differentially expressed genes (DEGs) among distinct RNA modification clusters were collected using “limma” package of R software. Next, we performed univariate cox regression model to analyze the correlation of each gene with overall survival, and the significant prognosis DEGs were used for further analysis. Subsequently, distinct genomic subtypes were determined by unsupervised clustering analyses. In addition, the prognostic analysis was performed for each genomic subtype and extract principal component 1 and 2 as the signature scores. Finally, the RNA modification score was defined using a method similar to that used in analyzing gene-gene interactions (GGIs): WM-Score = Σ (PC1i + PC2i), which is defined as the expression of final RNA modification phenotype-related genes (Sotiriou et al., 2006; Zeng et al., 2019).

### Statistical Analysis

A Wilcoxon rank-sum test was utilized to compare differences between two groups, and Kruskal-Wallis test was used for comparisons of multiple groups. The discrimination accuracy of the WM-Score model was described by receiver operating characteristic (ROC) analysis. Kaplan-Meier method estimate curves were generated for prognostic analysis, and the differences between groups were evaluated by a log-rank test. Univariate and multivariate analyses were further carried out to assess independent risk factors. All data were analyzed by the R 4.0.1 software. A two-tailed *p* < 0.05 was considered as statistically significant.

## Results

### Landscape of Genetic Alterations of 26 RNA Modification “Writers”

**FIGURE 1:**
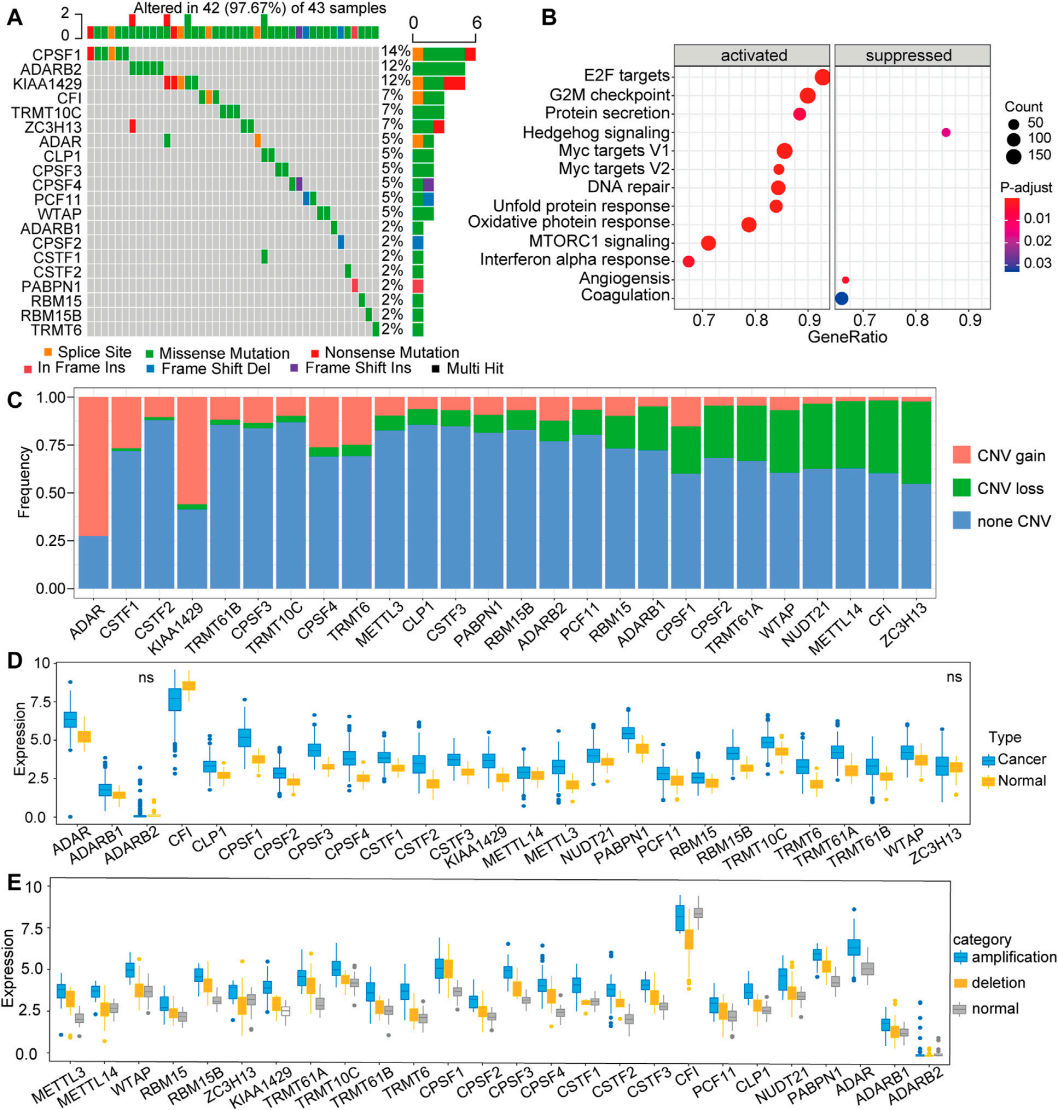
Expression pattern of 26 RNA modification “writer” genes in TCGA-LIHC **(A)** Frequency of mutations of the 26 identified regulator genes **(B)** Gene set variation analysis (GAVA) was used to compare the regulator mutation group and the non-mutation group **(C)** The CNV mutation frequency of 26 regulator genes in TCGA-LIHC **(D)** The expression of 26 RNA modification regulator genes between tumor tissues and control tissues **(F)** The mRNA expression of “writer” among three groups, including amplification group, CNV deletion group, and normal group.

A total of 26 RNA modification “writers” were selected in this study, which included seven m^6^A modification “writers”, three A-I modification “writers”, 12 APA modification “writers”, and four m^1^A modification “writers” (Supplementary Table S1) (Li et al., 2016; Tang S. J. et al., 2020; Shen et al., 2021). To explore the genetic alterations in RNA modification writers, we examined the incidence of somatic mutations and somatic copy number variation (CNV) for all “writers” based on the TCGA database. Among 356 samples from TCGA-LIHC, 42 (11.8%) exhibited genetic changes of these writers, and the details was shown in the Figure 1A. The highest mutation frequency was presented in CPSF1, followed by ADARB2 and KIAA1429 (Figure 1A), while METTL3, METTL14, TRMT61A, TRMT61B, CSTF3, and NUDT2 did not show any mutations in tumor samples. Next, we used the hallmark gene set to perform gene set variation analysis (GAVA) to compare the mutation groups and those without mutation in “writers”. The GSVA indicated significantly enriched carcinogenic activation pathways in the mutation group, such as those of E2F targets, G2M checkpoint, MYC, and MTORC1 signaling pathway (Figure 1B). Furthermore, the investigation of CNV alteration in 26 regulators showed that ADAR, CPSF1, CPSF4, TRMT10C and KIAA1429 had a widespread frequency of CNV gain, while ZC3H13, CF1, METTL14, NUDT21, and WTAP had a significant CNV loss (Figure 1C). To explore whether the above CNV alterations affected the expression of the 26 RNA modification regulators, we compared the expression level of these regulators between tumor samples and paired normal samples. The results showed increased mRNA levels of most “writers” in tumor samples in comparison to normal samples (Figure 1D), suggesting that CNV might be the major factor leading to the aberrant expression of medication regulators. Notably, the mRNA levels of some “writers” were increased, while the frequencies of CNV loss for those were high. Therefore, further investigations were performed. According to the CNV value, patients were divided into 3 groups, including CNV amplification group, CNV deletion group, and normal group, and the mRNA expression of “writers” were compared between these groups (Figure 1E). The results showed mostly elevated expression for the group of patients with CNV amplification compared with the other groups with CNV deletion or normal CNV in these “writers”. Taken together, we mapped the genetic alterations of the 26 RNA modification “writers” between control tissues and tumor tissues, suggesting that these changes might play vital functions in HCC tumorigenesis and progression.

### The RNA Modification Patterns Are Characterized by Distinct TIME Cell Infiltration Characteristics

**FIGURE 2:**
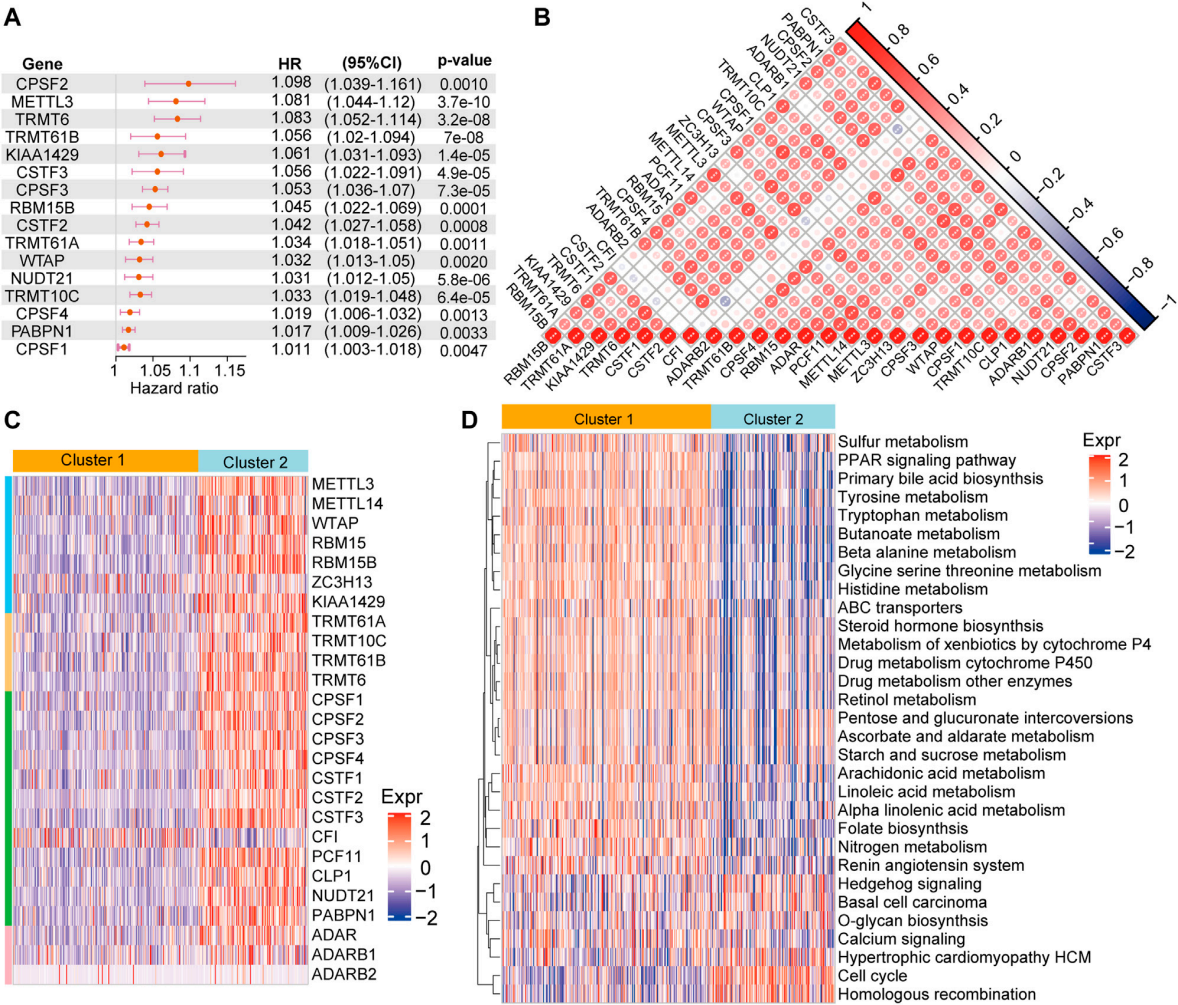
RNA methylation modification pattern and related biological pathways **(A)** The univariate cox regression analysis shows that 16 of 26 regulators are associated with of patient prognosis in the TCGA-LIHC cohort **(B)** Heatmap of the Spearman’s correlation analysis presenting negative (blue) and a positive (red) correlation among the “writers” in HCC **(C)** Unsupervised cluster analysis of 26 “writers” in HCC. Blue represents low expression of “writers” genes; red represents high expression of these genes **(D)** GSVA enrichment analysis of KEGG pathway chances between cluster 1 and 2. Blue indicate not activated pathways, and red indicates activated pathways.

In order to further understand the role of RNA modification “writers” in HCC, we performed univariate analysis of the 26 regulators based on the TCGA-LIHC cohort. We found that 16 of 26 “writers” were markedly correlated with the OS of HCC patients (Figure 2A). Next, we explored the relationship among “writers” and found that most were positively or negatively correlated with each other (Figure 2B). Thus, it is suspected that the crosstalk between different “writers” may have a vital function in the different modification patterns of HCC.

We performed consensus clustering to classify patients into distinct RNA modification patterns based on the mRNA expression of “writers” (Supplementary Table S2). Eventually, two RNA modification patterns with 204 cases were determined in pattern 1 (cluster 1), and 113 cases in pattern 2 (cluster 2) (Figure 2C). Subsequently, “GSVA” enrichment analysis was employed to further understand the biological behaviors between the distinct two clusters. Our results indicated that cluster 1 was significantly enriched in metabolism and drug metabolism pathways, such as sulfur metabolism, primary bile acid biosynthesis, tyrosine metabolism, tryptophan metabolism, drug metabolism cytochrome P450, drug metabolism other enzymes, renin angiotensin system, while cluster 2 enrichment pathways were mainly linked to proliferation and signal transduction, including cell cycle, calcium conduction, etc. (Figure 2D).

**FIGURE 3:**
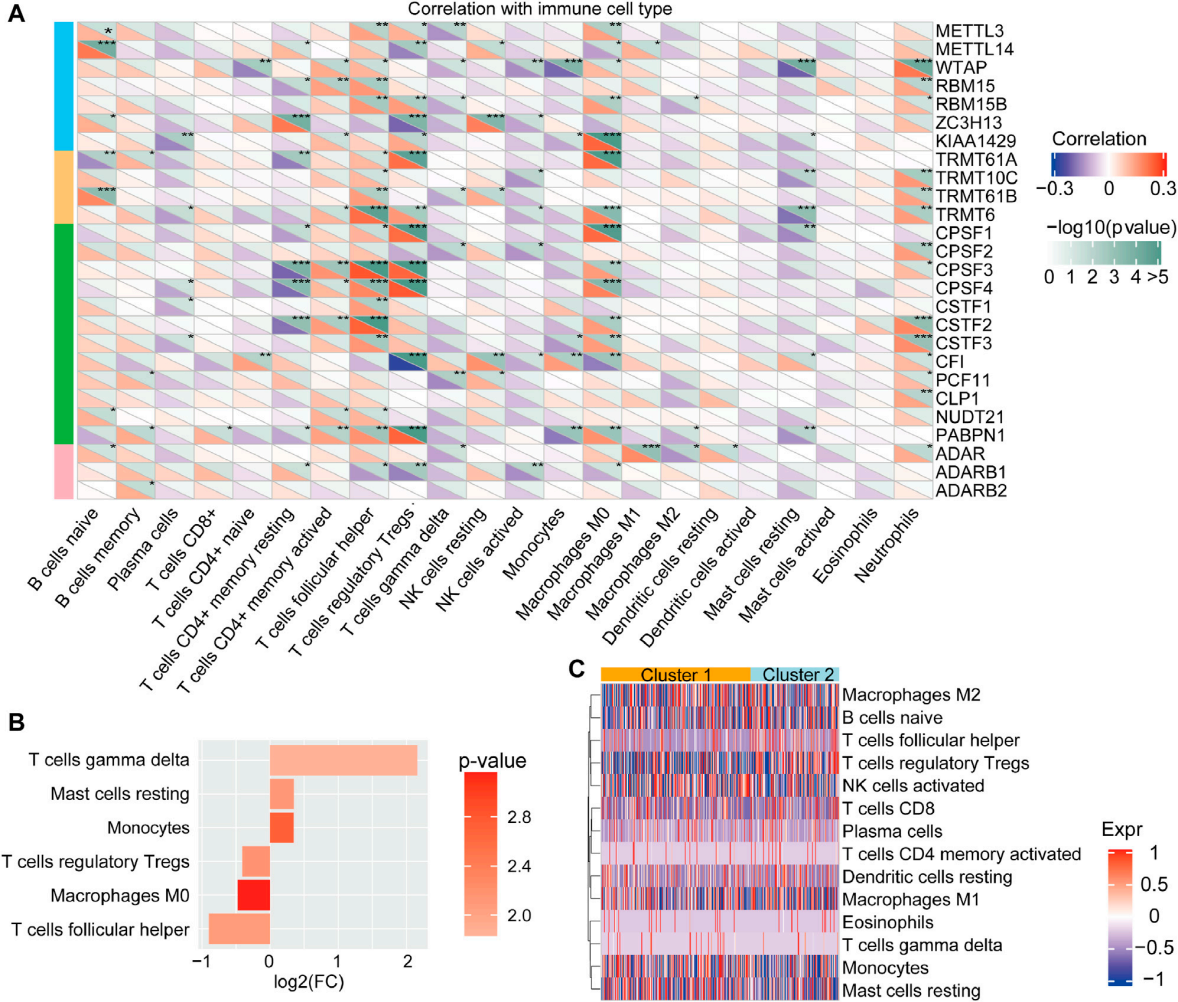
Tumor immune microenvironment characterization of the RNA modification patterns **(A)** The correlation between the 26 “writers” and TME in HCC was analyzed by CIBERSORT **(B)** The different characters of immune cell infiltration between clusters. Log(FC) > 0 represents that the immune cells were enriched in cluster 1 **(C)** The types of immune cells between distinct RNA modification patterns.

Emerging evidence suggests that RNA modifications interact with the tumorigenic environment, thus affecting tumor occurrence, development, and prognosis (Jiang et al., 2020; Chen H. et al., 2021; Chong et al., 2021). Therefore, the function of the RNA methylations in the TME were further explored. The association analysis using the CIBERSORT method revealed that the identified RNA modification regulators might have close links with immune cell infiltration from the TME (Figure 3A). For instance, METTL14, ZC3H13, CSTF3, and ADAR were markedly negatively associated with Mo macrophage differentiation, while their positive association was observed with METTL3, RBM15B, KIAA1429, TRMT61A, TRMT6, CPSF1, and NUDT21. Moreover, we analyzed the difference in immune cell infiltration from TME between cluster 1 and cluster 2. The results revealed that the infiltration of M2, T cells, mast cells, and monocytes was higher in cluster 1. Notably, though, the infiltration of M1, regulatory T cells and follicular helper T cells was higher in cluster 2 (Figures 3B,C). Overall, cluster 2 was usually enriched in immunosuppressive cells, indicating a poor prognosis, whereas cluster 1 was characterized by immune cell activity, indicating a beneficial prognosis. These findings suggested that RNA modification “writers” play crucial roles in immune cell infiltration and TME formation.

### Generation of RNA Modification Signature Model

**FIGURE 4:**
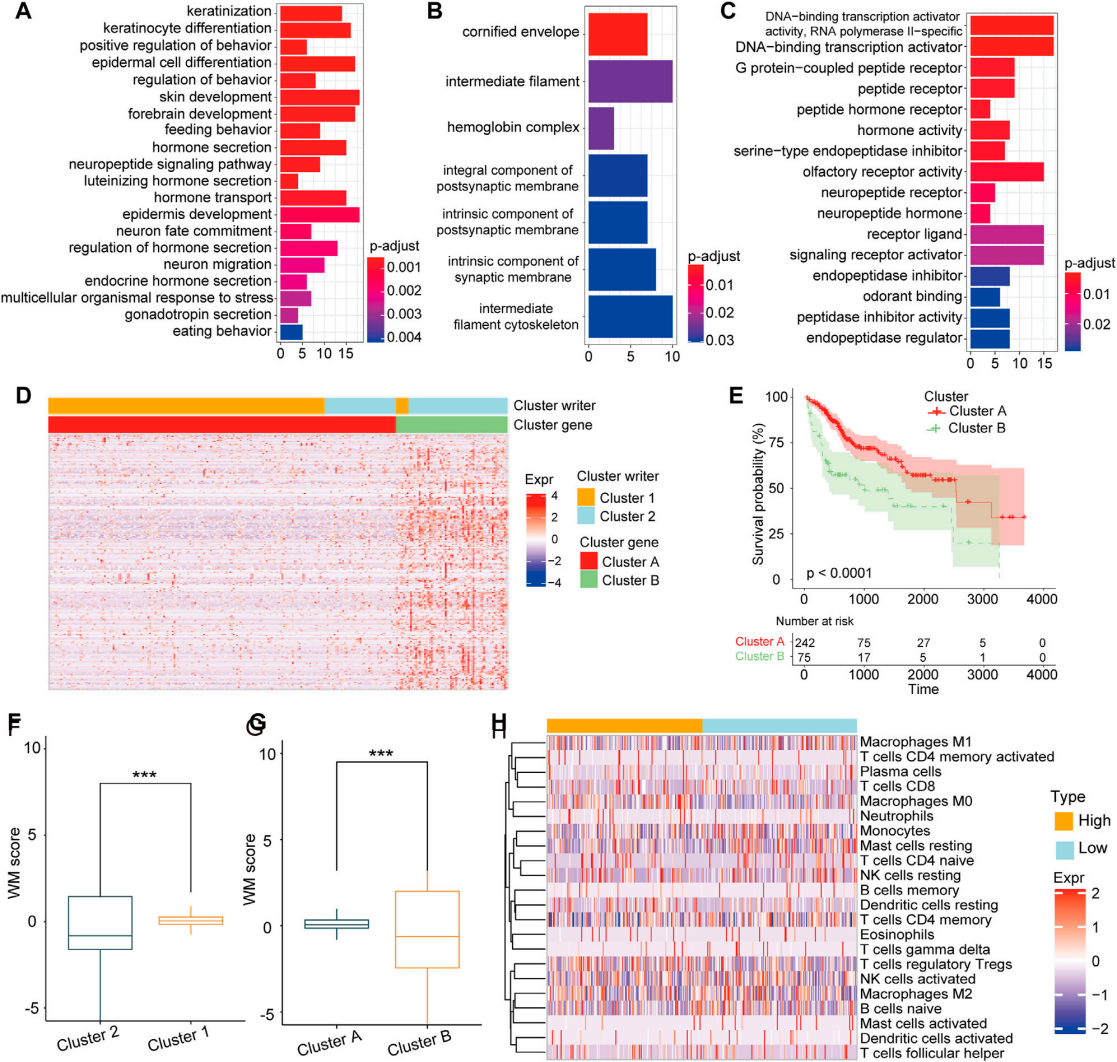
Construction of RNA modification model **(A–C)**. GSVA enrichment analysis revealed the DNA transcription signaling pathways. Stress reception signaling pathways **(A)** and signal transduction activation signaling pathways **(B)** were correlated with **(C)** 273 DEGs between cluster 1 and cluster 2 **(D)**. Unsupervised clustering of the 273 DEGs to identify two genomic subtypes **(E**) Survival analysis showing the poor prognosis of patients in cluster A group compared with those in cluster B group (*p* < 0.0001, Log-rank test) **(F)**. The score of cluster 1 was higher than that of cluster 2. **G**. The score of cluster A was significantly higher than that of cluster B **(H)**. The difference of immune cell infiltration abundance between WM-Score groups calculated by the CIBERSORT algorithm.

Our results above demonstrated the important role of RNA modification in TME formation and patient prognosis, while these findings were based on RNA modification patterns and could not accurately evaluate the capacity of the RNA modification as a prognostic predictor in individual HCC patients. The underlying genetic alterations in these two RNA modification patterns were still unclear. Based on these queries, we examined the transcriptional expression change between the two patterns. A total of 273 DEGs related to RNA modification patterns were identified, and the further enrichment analysis showed that these DEGs were enriched in many essential biological processes, including DNA-binding transcription activator activity, signaling receptor activator activity, and multicellular organismal response to stress (Figures 4A–C). Subsequently, according to unsupervised clustering analysis based on the 273 DEGs, patients were classified into two stable transcriptomic subtypes: cluster A and cluster B (Figure 4D), with 242 and 75 of the 317 HCC patients, respectively. The prognosis of patients in cluster B was poorer than those in gene cluster A (Figure 4E; *p* < 0.0001, log-rank test).

Furthermore, we developed a score model based on the DEGs between gene clusters. As described in the Methods section, a scoring model named writers of RNA modification-score (WM-Score) was constructed. We discovered that cluster 1 had a higher WM-Score value than cluster 2 (Figure 4F). Consistently with this, cluster A also showed a higher score value than cluster B (Figure 4G). To evaluate the association of WM-Score value with TME, we further calculated the abundance of immune cell infiltration for the low and the high WM-Score value groups. We found that the infiltration rate of M0 macrophages, monocytes, and TfCD8 was higher in the high WM-Score value group, and that of activated NK-activated cells and M1 macrophages was higher in the low WM-Score value group (Figure 4H).

### Association Between WM-Score and Clinical Characteristics

**FIGURE 5:**
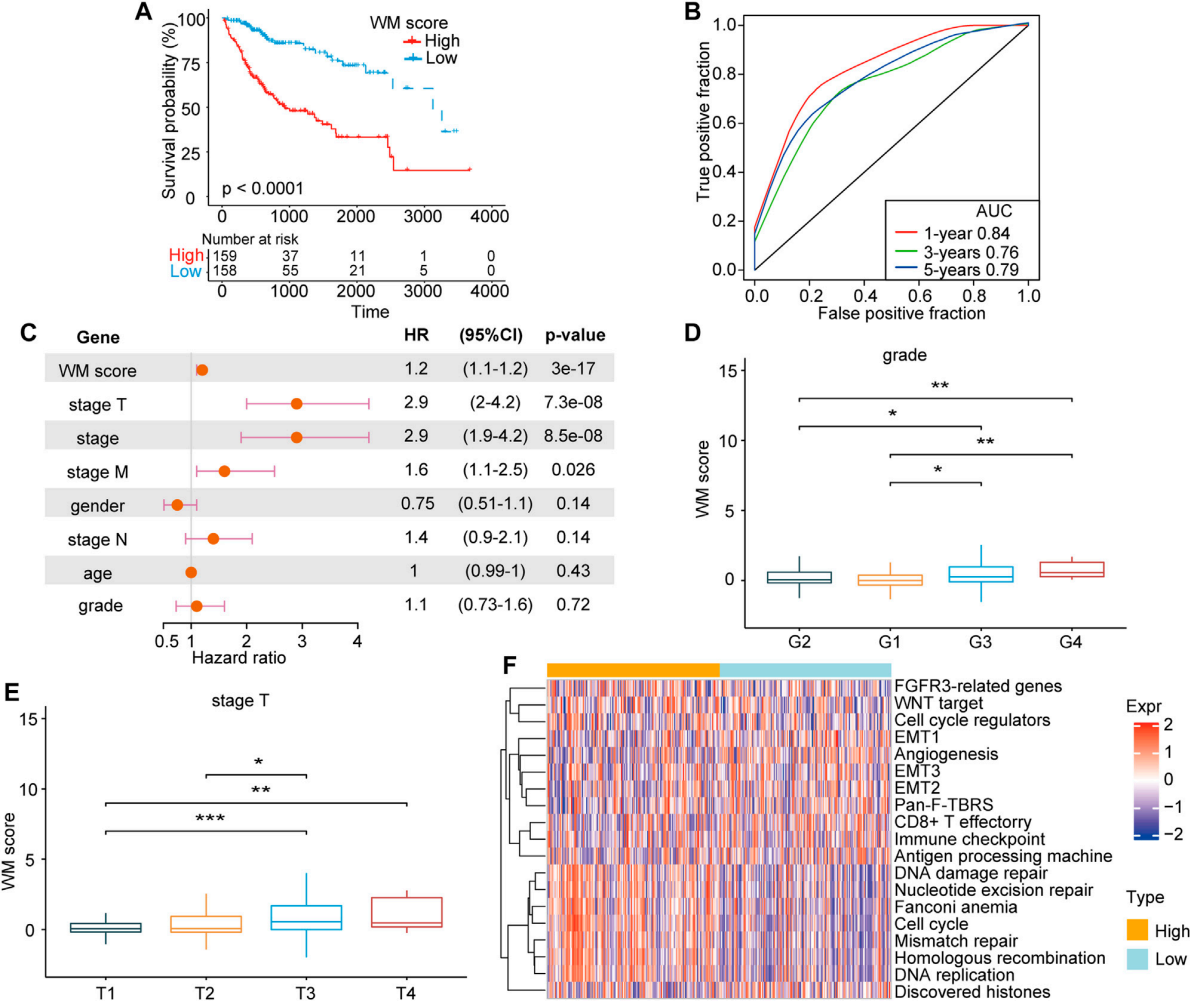
The clinical characteristics and prognosis of HCC correlated with the WM-Score model. **(A)**. Kaplan-Meier overall survival for HCC patients in the high and low WM-Score groups. **(B)**. The predictive accuracy of the WM-Score model in the TCGA-LIHC cohort (AUC: 0.84, 076, and 0.79, corresponding to 365, 1,095, and 1825 days OS, respectively). **(C)**. Multivariate cox regression analysis of factors, which included WM-Score, stage-T, stage, stage-M, gender, stage-N, patient age, and grade in the TCGA-LIHC cohorts. **(E)**. WM-Score differences among grade and stage-T of HCC in TCGA-LIHC. **(F)**. Heatmap showing the GSVA score of EMT signaling pathways between different WM-Score groups in TCGA-LIHC.

After confirming the efficacy of the WM-Score model in predicting patient prognosis, we investigated whether this model could be applied to determine the tumorigenesis, progression, invasion and metastasis of HCC. The prognostic efficiency of the scoring model was explored through classifying patients into low and high score groups the using “survminer” package. As expected, patients with high score demonstrated a poorer prognosis than those with low score in the TCGA-LIHC cohort (Figure 5A). We used ROC curve analysis to determine the discrimination accuracy of the scoring model in predicting patient prognosis. The area under the ROC curves (AUCs) of WM-Score values were 0.84, 0.76 and 0.79 at 1, 3 and 5 years overall survival, respectively (Figure 5B). Multivariate analysis for the TCGA-LIHC cohort also demonstrated that the WM-Score could serve as an independent prognostic predictor in HCC (Figure 5C). All of these results indicated that the WM-Score model has accurate prognostic value for HCC patients. The analysis of difference in WM-Scores between different TNM grades and clinical grades in the TCGA database indicated that samples with higher clinical grades and TNM stages usually have higher WM-Score values (Figures 5D,E). In addition, considering the EMT-related pathways, the samples with different WM-Score value had different pathway characteristics. For the TCGA database, samples with high WM-Score value were significantly related to cell cycle, DNA damage repair, and DNA replication, while samples with low WM-Score value were related to EMT, WNT target, and cell cycle regulators (Figure 5F).

### Value of WM-Score Model in Chemotherapy and Therapy Sensitivity

**FIGURE 6:**
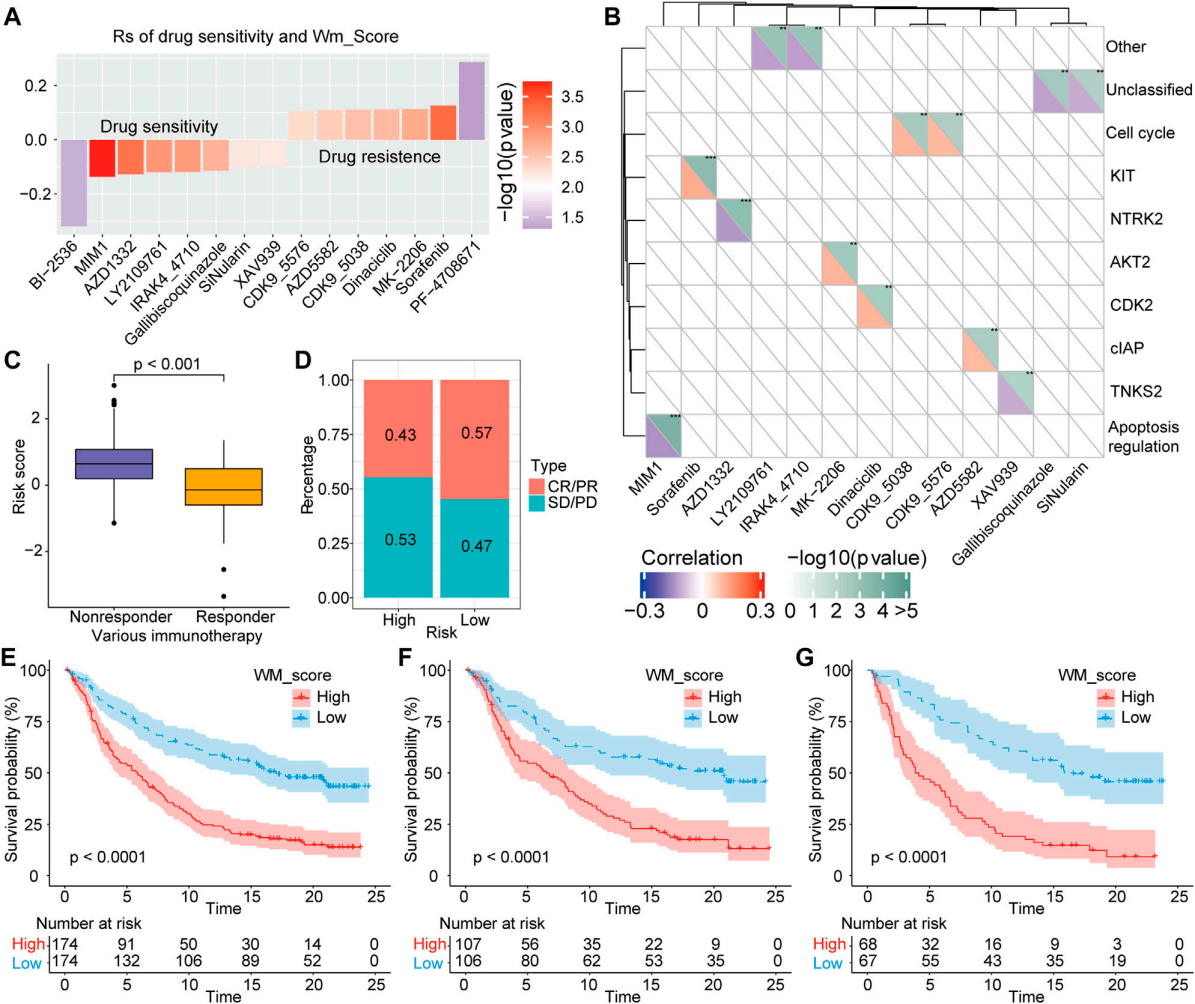
Correlation of scoring model with drug reaction and immunotherapeutic response. **(A)**. Spearman’s analysis was used to determine the correlation between score and drug response in GDSC. **(B)**. The association between drugs and targeted signaling pathways. **(C)**. The difference in the score between immunotherapeutic responder in the IMvigor210 cohort. **(D)**. The percentage of patients with different responses (including SD, PD, CR, and PR) to PD-L1 blockade immunotherapy. E-G. Total samples, or Stage I + II samples, or Stage III + IV samples in the IMvigor210 cohort all showed a significant difference in survival between samples with high and low WM-Scores based on survival analysis.

For several years, sorafenib has been approved a treatment option for advanced HCC patients, while efficacy of sorafenib is limited by drug resistance (Gnoni et al., 2019). Aiming to further investigate whether the WM-Score value affected drug sensitivity, we evaluated the correlation between the scoring model and the drug response of tumor cell lines. Using Spearman’s correlation analysis, 15 significant correlation pairs were identified in the Cancer Drug Sensitivity Genomics (GDSC) database between scoring model and drug reaction (Yang et al., 2013). Among them, eight pairs of drug sensitivity were related to WM-Score value, and seven pairs showed resistance related to WM-Score value (Figure 6A). In addition, we also analyzed the signaling pathways of these drugs to determine target genes. We found that drugs associated with high WM-Score value mainly target KIT, CLAP, and cell cycle signaling pathways. In contrast, drugs related to low WM-Score value mostly target apoptosis regulation and cell cycle signaling pathways (Figure 6B). Taken together, these findings indicate that the WM-Score values are related to drug reaction, and thus might offer a framework to guide the treatment strategy of HCC.

### Role of WM-Score Model in Predicting anti-PD-1/L1 Immunotherapy

In recent years, immune checkpoint inhibitors (ICIs) have made breakthroughs in the treatment of advanced HCC, while biomarkers that could effectively predict the efficacy of immunotherapy are still lacking. Herein, we explored whether the WM-Score model could predict therapeutic response to ICI therapy in HCC patients. For the IMvigor210 cohort, the therapeutic efficacy was significantly better in patients with low WM-Score value compared to those with high WM-Score value (Figure 6C). The frequency of response to anti-PD-1/L1 treatment in the low WM-Score value group was higher than that in the high WM-Score value group (Figure 6D). We also analyzed the survival difference of all samples of IMvigor210 and those under different stages. The results showed that total samples (Figure 6E), or Stage I + II samples (Figure 6F), or Stage III + IV samples (Figure 6G) all exhibited a marked difference in survival between samples with high and low WM-Score value. Especially in the prediction of high-stage clinical samples, the WM-Score value demonstrated extremely high power. Collectively, our results proved that the WM-Score model might serve as a potential predictor of response to anti-PD-1/L1 immunotherapy.

## Discussion

A growing pool of evidence indicates that RNA modifications play a key role in gene expression, whose disruption impacts the pathogenesis of human disease, including cancer (Frye et al., 2016). Although RNA modifications as genetic or epigenetic alterations of genes are not traditionally considered as cancer drivers, cumulative evidence suggests that abnormal RNA modifications are functionally correlated with many hallmarks of cancer, such as proliferation, invasion, migration, differentiation, self-renewal, and response to therapy (Cui et al., 2017; Weng et al., 2018; Jin et al., 2019).

For instance, N6-methyladenosine (m^6^A) is an RNA methylation that is the most abundant form of internal mRNA modification. Yang et al. reported the involvement of the m^6^A modification in the 3′-UTR of oncogene CDCP1 mRNA in bladder cancer cell growth and progression (Yang et al., 2019). Lang et al. indicated that the m^6^A modification showed an important function in regulating the stability of viral transcripts and EBV-mediated tumorigenesis (Lang et al., 2019). Furthermore, Lan et al. reported that m6A methyltransferase KIAA1429 was high expressed in HCC tissues and knockdown KIAA1429 inhibited cell proliferation and metastasis *in vitro* and *in vivo* (Lan et al., 2019). Chen et al. found the writer CPSF1 of APA was significantly increased in HCC tissues and associated with poor survival outcomes (Chen S.-l. et al., 2021). All these studies focused on one or two modification regulators to explore their dysregulation, function, and underlying mechanism in cancer, however, the deposition of RNA modifications is a dynamic process involving multiple modification regulators. In the present study, we comprehensively described the molecular and biological features of different regulators of RNA modifications and identified two distinct RNA modification subtypes based on multiple modification regulators. Importantly, the two subtypes (cluster 1 and cluster 2) are not only associated with clinical survival, but also with the abundance of immune cell infiltration.

Considering the diversity and complexity of TME, the thorough understanding of its implications in cancer is a significant challenge. In recent years, some research groups have documented that RNA modifications were closely associated with TME. Shen et al. attempted to explore the role of m^6^A regulators in HCC immune cell infiltration and prognosis, and identified three m^6^A subtypes based on TCGA and GEO database, which were related to three known immune phenotypes (including immune-inflamed phenotype, immune-excluded phenotype, and immune-desert phenotype) (Shen et al., 2021). Chong et al. also discovered three m^6^A modification patterns among 1,370 colon cancer cases, which were correlated with different outcomes and TME characterization (Chong et al., 2021). Three m^6^A modification patterns with distinct TME cell-infiltrating characteristics were also determined in gastric cancer (Zhang et al., 2020), lung adenocarcinoma (Li et al., 2020), pancreatic adenocarcinoma (Tang R. et al., 2020), and gliomas (Xu et al., 2020). Similar with our analysis, these studies were based on a large number of samples in the subject database, such as TCGA and GEO, in order to clarify the role of modification in tumor immune regulation and progression. In our study, we further identified two stable transcriptomic subtypes based on the DGEs of the two RNA modification clusters. Especially, the transcriptomic subtypes were significantly associated with the immune cell activation and prognosis of HCC patients. Thus, the systematical evaluation of RNA modification patterns provides novel clues for understanding TME characterization in HCC. Gu et al. found 3 m 5C regulator-mediated methylation modification patterns based on the expression of 13 m 5C regulators which were closely associated with different immune cell infiltration characteristics in HCC (Gu et al., 2021). Shen et al. demonstrated three m6A modification patterns which affect tumor immune infiltrates and prognosis of patients with HCC (Shen et al., 2021). Previous studies mainly centered upon one types of RNA modification to explore their effect on TME. Here, we performed a comprehensive analysis of multiple types of RNA modification and highlights the cross-talk and the roles of RNA modifications in the TME and response to immunotherapy.We developed the WM-Score model to accurately predict the prognostic value of the RNA modification in individual patients. We found that this model could be applied to assessing clinicopathological features, such as clinical grades and TNM grades, and patients with higher clinical grades and TNM grades usually had higher WM-Score value.

In addition, the RNA modification pattern with higher WM-Score value tended to correlate with immune cell suppression in the tumor microenvironment, while the pattern with lower WM-Score value was usually associated with immune activation. In the IMvigor210 cohort, WM-Scores model was found to be linked with immune cell infiltration in TME as well as response to anti-PD-1/L1 immunotherapy, suggesting the application potential of WM-Score model for predicting HCC anti-PD-1/L1 immunotherapy.

## Conclusion

In the present work, the RNA modification regulators were comprehensively analyzed, and the correlation was demonstrated between RNA modification patterns and cell-infiltrating characteristics in the TME. The systematic evaluation of individual tumor RNA modification pattern might serve as a useful predictor of prognosis for HCC patients and act as a valuable tool for developing more effective immunotherapy strategies.

## Data Availability

The original contributions presented in the study are included in the article/Supplementary Material further inquiries can be directed to the corresponding author.
